# Individualized recombinant human thrombomodulin (ART-123) administration in sepsis patients based on predicted phenotypes

**DOI:** 10.1186/s13054-019-2521-7

**Published:** 2019-06-24

**Authors:** Daisuke Hasegawa, Osamu Nishida

**Affiliations:** Department of Anesthesiology and Critical Care Medicine, Fujita Health University School of Medicine, 1-98 Dengakugakubo, Kutsukake-cho, Toyoake, Aichi 470-1192 Japan

Letter to the editor

Disseminated intravascular coagulation (DIC) is a relatively common complication of sepsis. The pathophysiology of septic DIC is associated with neutrophil extracellular trap (NET) formation [1] which results in subsequent thrombi formation known as immunothrombosis, especially in the liver. There are at least two known types of NETs; one type known as vital NETs is produced directly by activation of neutrophils on invasion by pathogens. The other type known as suicidal NETs is produced indirectly through the subsequent over-production of cytokines [2]. Vital NETs, which develop as an initial response to the pathogen, are shown to be suppressed by recombinant human thrombomodulin (ART-123), while suicidal NETs are not [1]. Therefore, ART-123 is potentially more effective when it is administered during the early phase of sepsis. In a phase 3 randomized controlled trial, ART-123 failed to show any differences in 28-day mortality compared to placebo [3]. In this trial, only patients with pre-existing coagulopathy were included based on the post hoc analysis of a phase 2 trial which had shown survival difference in this specific subgroup population. It is possible that the administration of ART-123 was too delayed to work effectively, since only patients with pre-existing coagulopathy were included and suicidal NETs were likely already activated in this population. Therefore, it is crucial to determine the cases that will benefit most from ART-123 treatment during the early phase.

There is increasing interest in the classification of sepsis [4] for personalized treatment options. More recently, a large, retrospective analysis based on machine learning method classified phenotypes of patients with sepsis into four clinical types (α, β, γ, and δ) that correlated with host response patterns using demographic data, physical examination findings, and laboratory data on arrival [5]. Of these, patients with the δ phenotype—characterized by liver dysfunction and shock—were more inclined to develop coagulopathy and had a higher mortality rate than those of patients with other phenotypes. Based on the findings of both studies, we hypothesize that patients with this phenotype are potentially most likely to benefit from ART-123. Since the phenotypes can be obtained before the patients develop coagulopathy, ART-123 can be administered during the early phase for those who are expected to develop coagulopathy. Further clinical trials with earlier intervention based on the clinical phenotyping for precision medicine are warranted to demonstrate mortality benefit of this theoretically effective medication for septic DIC.

## Data Availability

Not applicable.

## Abbreviations

DIC: Disseminated intravascular coagulation
NETs: Neutrophil extracellular traps

## References

[1] Shimomura Y, Suga M, Kuriyama N, Nakamura T, Sakai T, Kato Y (2016). Recombinant human thrombomodulin inhibits neutrophil extracellular trap formation in vitro. J Intensive Care.

[2] Yipp BG, Kubes P (2013). NETosis: how vital is it?. Blood..

[3] Vincent Jean-Louis, Francois Bruno, Zabolotskikh Igor, Daga Mradul Kumar, Lascarrou Jean-Baptiste, Kirov Mikhail Y., Pettilä Ville, Wittebole Xavier, Meziani Ferhat, Mercier Emmanuelle, Lobo Suzana M., Barie Philip S., Crowther Mark, Esmon Charles T., Fareed Jawed, Gando Satoshi, Gorelick Kenneth J., Levi Marcel, Mira Jean-Paul, Opal Steven M., Parrillo Joseph, Russell James A., Saito Hidehiko, Tsuruta Kazuhisa, Sakai Takumi, Fineberg David (2019). Effect of a Recombinant Human Soluble Thrombomodulin on Mortality in Patients With Sepsis-Associated Coagulopathy. JAMA.

[4] Zhang Z, Zhang G, Goyal H, Mo L, Hong Y (2018). Identification of subclasses of sepsis that showed different clinical outcomes and responses to amount of fluid resuscitation: a latent profile analysis. Crit Care.

[5] Seymour Christopher W., Kennedy Jason N., Wang Shu, Chang Chung-Chou H., Elliott Corrine F., Xu Zhongying, Berry Scott, Clermont Gilles, Cooper Gregory, Gomez Hernando, Huang David T., Kellum John A., Mi Qi, Opal Steven M., Talisa Victor, van der Poll Tom, Visweswaran Shyam, Vodovotz Yoram, Weiss Jeremy C., Yealy Donald M., Yende Sachin, Angus Derek C. (2019). Derivation, Validation, and Potential Treatment Implications of Novel Clinical Phenotypes for Sepsis. JAMA.

